# Measurement bruise volume of olive during impact test using FEM and quality evaluation of extracted olive oil

**DOI:** 10.1002/fsn3.2508

**Published:** 2021-08-03

**Authors:** Mahdi Rashvand, Abbas Akbarnia, Rouzbeh Abbaszadeh, Davoud Karimi, Ali Jafari

**Affiliations:** ^1^ Machine design and Mechatronics Department Institute of Mechanics Iranian Research Organization for Science and Technology (IROST) Tehran Iran; ^2^ Biosystem Engineering Department Agricultural Research Institute Iranian Research Organization for Science and Technology (IRSOT) Tehran Iran; ^3^ Faculty of Agriculture Machinery, Agriculture and Natural Resources Department Tehran University Tehran Iran

**Keywords:** bruise, finite element method, olive oil, quality, stress

## Abstract

Mechanical damage is a phenomenon that always occurred in the postharvest process. Due to the inappropriate harvest and postharvest process of Olive that lead to the bruise phenomenon, the quality of the extracted olive oil reduces. In this study, the effect of olive damage on bruising volume and quality characteristics was investigated. Three different varieties of Yellow, Oily, and Fishemi in three stages of unripe, semiripe, and ripped were used. Also, three kinds of the surface of rubber, nylon, and foam for the drop test were considered. The tests were performed in laboratory mode and simulated. For all tests, with increasing maturity, the amount of stress and internal energy were decreased and the bruise volume was increased. The amount of bruise volume and bruise susceptibility were obtained, and the experimental and simulated conditions were compared. On the other hand, the quality characteristics of olive oil including free fatty acid (FFA), peroxide value (PV), k232 and K270 coefficients, total chlorophyll, total carotenoid, total phenol, and total flavonoid were measured. The results showed that the finite element and chemometric methods are acceptable methods for predicting the generated energy of the fruit during impact, the amount of bruising volume, as well as evaluating the quality of the extracted oil.

## Graphical abstract

Deformation behavior of three olive varieties with three ripening stage was obtained. Finite element analysis based on explicit dynamic simulations was conducted. The bruise volume of measured and simulated models for all types of cases was compared. Chemical evaluation of damaged olive oil samples was performed.

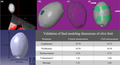

## 1 INTRODUCTION

Mechanical damage of fruit is one of the consequences of inappropriate harvesting and postharvest process (Lu & Lu, 2017). Nowadays, the harvesting of agriculture products is mechanized by different harvesting machines and then transferred to food industry for postharvest operations (Komarnicki et al., 2017). The shape of damage depends on the physical and biological structure of the product as well as the type of load (static, dynamic, and oscillating). These occurrences sometimes damage the internal tissue of the fruit which not only spoils the fruit tissue but also leads to discharging the material of the inside cell and spreading damage (Li et al., 2017).

Usually, bruising caused by the impact is not detectable immediately and depending on the flesh and biological characteristics of the fruit, it is observable after a few hours (Scheffler et al., 2018). On the other hand, the water and much carbohydrate content which contribute to the metabolic processes lead to an increasing bruised area. This damage of fruit's skin remains even after the fermentation process. Phenolic compounds with the activity of polyphenol oxidase (PPO) and peroxidase (POD) play important roles in damaged tissues (Bugaud et al., 2014). Therefore, the phenomenon of bruising during harvesting operation or postharvesting of agriculture products should be considered.

The bruise phenomenon has an impact on the quality of the olive oil which extracted from the damaged olive. Mechanical damage of olive during harvest time and postharvest lead to enhancing the oxidation process of the oil, thereby increasing the value of acidity and peroxide. Hence, the amount of volatile acids (acetic acid and butyric acid) increases and eventually causes an unpleasant odor in the oil. The fruits had less damaged regions have a higher amount of polyphenols and lower peroxide. Consequently, it is important to identify the mechanical damage and utilize appropriate methods to reduce the damage.

Many researchers investigate the correlation of bruising level to various mechanical factors such as force, drop height, impact velocity, and absorbed energy (Ahmadi et al., 2010; Jiménez et al., 2016; Jiménez‐Jiménez et al., 2013; Yousefi et al., 2016; Zarifneshat et al., 2010). With the advancement of technology, some researchers suggested to use simulation method to predict the effect of impact such as bruising (Abedi & Ahmadi, 2014; Celik, 2017; Du et al., 2019; Kabas, 2010).

The finite element method can be used to solve complex engineering subjects and analyze the stress of the loaded object. Issues that cannot be solved by simple analytical methods or require costly, time‐consuming, and destructive experiments can solve using FEM (Ahmadi et al., 2016; Khorsandi et al., 2017). Some subjects require nonlinear analysis to achieve more accuracy. Many researchers used the explicit method for solving complex issues of impact and contact (Celik, 2017; Celik et al., 2017; Du et al., 2019; Zhao et al., 2019). Also, impact test is acceptable method to determine bruise volume and bruise resistance (Komarnicki, Stopa, Szyjewicz, et al., 2017). Geometry is another factor in obtaining more accuracy. Different 3D scanners were utilized to create cloud points of the solid shape and then modeled using CAD software.

Based on the mentioned points, the bruise that caused by the impact is one of the important results in olive harvesting as well as the postharvest process. The main aim of this study was to investigate the value of stress and bruise volume of olive during postharvest process and evaluation of effects of impact on the quality of the extracted olive oil.

## 2 MATERIAL AND METHODS

### 2.1 Measurement of physical and mechanical properties

**FIGURE 1:**
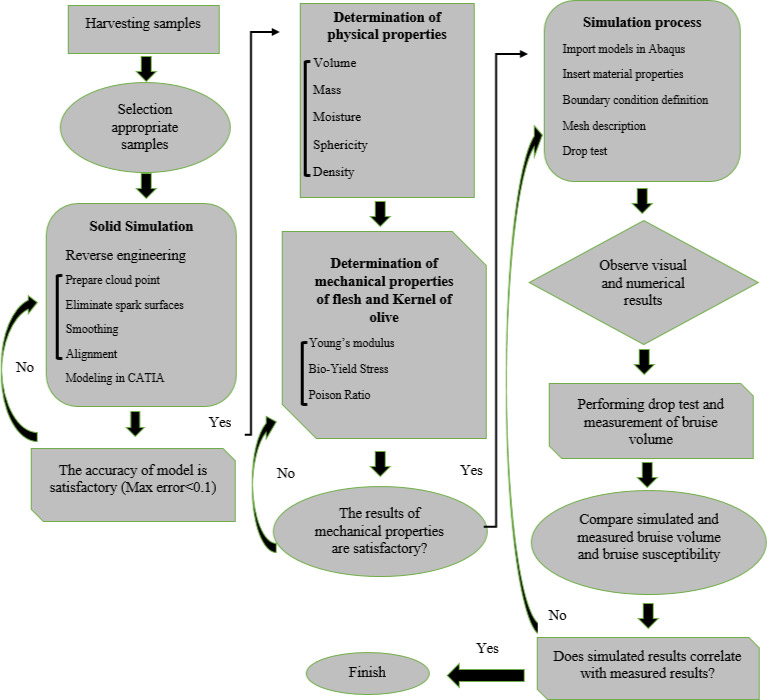
Research process diagram

Iran has different olive varieties in which most of them are cultivated in the north of the country. In this study, three types of main olive varieties of Yellow, Oily, and Fishemi were used. Since the physical and especially mechanical properties of olive fruit change during the time, the three stage of unripe (early October), semiripe (early November), and ripped (late November) was harvested. The trees were planted at a distance of 8 × 6 meters, and the trees were irrigated by drip method. Three trees with the same conditions were selected from each cultivar. Considering the class of each ripening stage, 5 samples with 4 replicates were selected. Totally, 20 intact samples were selected from each class to determine their physical and mechanical properties. The process diagram of this research is shown in Figure 1.

Physical properties of olive samples consist of dimensions (length, width, and thickness), mass, spherical coefficient, moisture, and density were measured using digital caliper Hornady 050,080 model, digital scale, inclined plane, psychomotor Farmex model—USA, and fluid displacement test, respectively. The average results of the different classes were reported in Table 1. The Yellow variety was larger than the other two cultivars at all ripening stages. Whereas, the mass of the Oily cultivar was greater than Fishemi and Yellow varieties. It seems the moisture factor affects the mass. The lower moisture content leads to more oil content in olive and can be one of the factors in the mass of the olive (Abasi et al., 2018; Yeow et al., 2010). Because the kernel of the olive varieties did not differ significantly, the physical characteristics of the kernels of 20 intact olive samples were measured. In order to modeling in the software just required volume (1.2 mm^3^), mass (0.9 g) and density (2.2 g.mm^‐3^) of the kernel.

**TABLE 1:** Average results of olive samples

Variety		Fishemi			Oily			Yellow		
Properties	Ripening stage	Unripe	Semiripe	Ripped	Unripe	Semiripe	Ripped	Unripe	Semiripe	Ripped
Volume (mm^3^)		3.87 ± 0.4	4.53 ± 0.3	5.2 ± 0.35	4.66 ± 0.4	5.43 ± 0.5	6.79 ± 0.2	5.4 ± 0.4	6.74 ± 0.45	9.03 ± 0.25
Mass (gr)		3.32 ± 0.6	4.63 ± 0.35	5.45 ± 0.4	4.49 ± 0.5	5.72 ± 0.6	6.37 ± 0.45	3.92 ± 0.3	5.47 ± 0.4	5.84 ± 0.35
Moisture (%)		54.55 ± 4	47.28 ± 6	46.1 ± 2	55.4 ± 7	43.07 ± 3	36.91 ± 5	61.56 ± 2	55.3 ± 4	40.78 ± 3
Sphericity (%)		63.61 ± 6	64.31 ± 4	65.19 ± 5	65.8 ± 4	66.24 ± 7	67.4 ± 5	64 ± 4	62.43 ± 6	64.92 ± 5
Density(g/mm^3^)		0.86 ± 0.08	1.02 ± 0.1	1.06 ± 0.1	0.97 ± 0.07	1.07 ± 0.9	0.95 ± 0.8	0.73 ± 0.05	0.84 ± 0.04	0.66 ± 0.4

**FIGURE 2:**
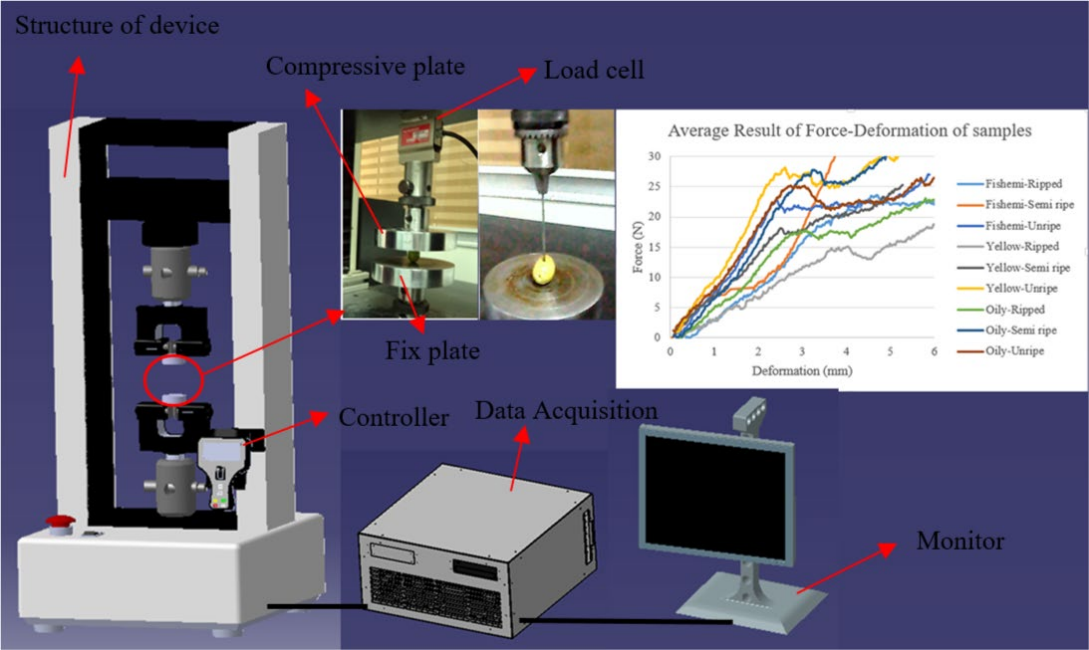
Compression apparatus Components and average of output plots

In the next step, to obtain the mechanical properties, the tensile‐compression test was performed by the Instron device (Santam Model‐Iran) at standard temperature (293 K). Loading was performed using two flat plate and probe methods (Figure 2). Based on the ASABE standard for agricultural materials, the best speed of the compressive plate is 2.5–30 mm/min. Most researchers used speed of 10 mm/min (or below) and frequency of 10 Hz (Ahmadi et al., 2016; Celik, 2017; Pieczywek & Zdunek, 2014). They obtained best results for bioyield points using mentioned speed. In this study, the speed of 5 mm/min, frequency of 10 Hz, and load cell of 50 N were used in the compression experimental. After loading, the deformation–force results were saved and the deformation–force curve was plotted. The average results of the test (using the flat surface) for each class are presented in Figure 2.

The force–deformation curve of the fruit can be modeled using Taylor's quadratic polynomial expansion. The coefficients of a, b, and C (Equation 1) represent three types of elastic, viscous, and fracture behavior, respectively, which was occurring simultaneously in the flesh of fruit.

(1) $$F=\text{aD}+{\text{bD}}^{2}+{\text{cD}}^{3}$$

The cubic spline of the force–deformation curve for all classes was fitted using Microsoft Excel software. The maximum apparent modulus and the tangential modulus were calculated using this curve. The slope at each point of the curve is the tangent modulus value. By differentiating the original equation, the tangential modulus was obtained (Equation 2). The maximum slope occurs at the inflection. At this point, there is a maximum resistance to deformation. The apparent modulus is slope value from the origin of the axis to any point on the curve (Equation 3). In order to determine model's coefficients for each sample, the cubic polynomial function of the force–displacement curve was fitted.

(2) $$T=a+2\text{bx}+3{\text{cx}}^{2}$$

(3) $$q=\frac{F}{x}=a+\text{bx}+{\text{cx}}^{2}$$

which *T* is bioyield point, *q* is value of deformation, *F* is force, x is shifted distance of plate, and a,b,c are Henry's coefficients.

**FIGURE 3:**
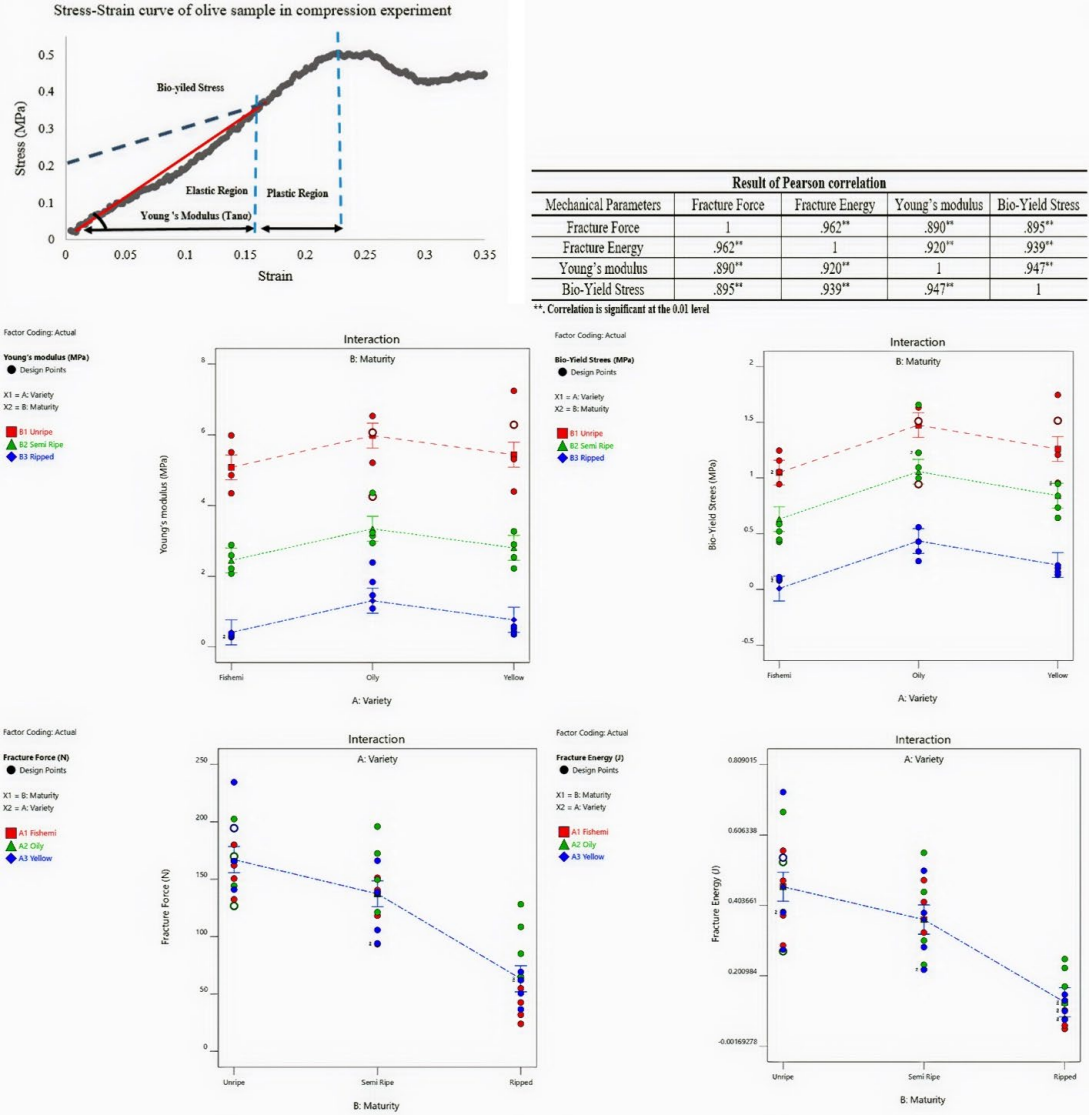
Mechanical properties of olive samples and Pearson test results

Mechanical properties include fracture energy, fracture force, modulus of elasticity, and yield stress. The maximum fracture energy and fracture force of the Yellow variety at the unripe stage were 0.761 J and 241 N, respectively, which was more than the other two cultivars. However, the resistance of the Oily variety at the semiripe and ripe stage was more than the Yellow and Fishemi cultivars. The maximum fracture energy of the Oily, Yellow, and Fishemi was 0.235, 0.152, and 0.110 J, and the maximum fracture force was 132.36, 71.63, and 59.73 N, respectively. Also, the elastic modulus and bioyield stress of samples were obtained. In addition, the relation between the fracture energy, fracture force, elastic modulus, and bioyield stress was presented using the R test and the Pearson method (Figure 3). All of the factors were significant at 0.01% level. The ripening stage plays a key role in the mechanical properties. The effect of the ripening stage of all samples on mechanical factors was investigated (Figure 3). Stress–strain curves were extracted from testing the mechanical properties of olives. In Figure 3, an example of the stress–strain curve of olive yellow cultivar was shown in the ripped stage. Depending on the shape, the deformation is initially elastic, and after reaching the bioyield point, it slowly deforms into permanent deformation.

In the simulation of the process, insert of proper mechanical properties of the fruit effect on the accuracy of the result. Table 2 presents the min, max, and average values of bioyield stress, Young's modulus factors, and Poisson's ration of classes. During ripening stages and due to the chemical activity inside the olive fruit (olive flesh), the resistance of the fruit to deformation decreases. The average value of Young's modulus of Yellow, Oily, and Fishemi varieties in the unripe stage was 5.81, 5.51, and 5.17 MPa, respectively. However, the maximum and average of Young's modulus and yield stress values of Oily cultivar were more than Yellow and Fishemi varieties in the semiripe and ripped stages. Also, the required mechanical parameters to simulate three surfaces of rubber, nylon, and foam were reported (Table 3).

**TABLE 2:** Mechanical parameters of the olive samples

Parameters	Variety	Fishemi			Oily			yellow		
		Unripe	Semiripe	Ripped	Unripe	Semiripe	Ripped	Unripe	Semiripe	Ripped
Young's modulus (MPa)	Min	4	2.01	0.26	3.85	2.85	0.95	4.05	2.15	0.33
	Max	6.26	3.01	0.39	6.78	4.82	2.57	7.52	3.37	0.59
	Ave	5.17	2.44	0.32	5.51	3.41	1.69	5.81	2.73	0.46
Bioyield Stress(MPa)	Min	0.86	0.41	0.07	0.85	0.96	0.22	0.88	0.62	0.12
	Max	1.30	0.61	0.11	1.68	1.83	0.60	1.81	0.97	0.22
	Ave	1.10	0.49	0.09	1.32	1.24	0.39	1.35	0.79	0.17
Poison Ratio		0.28								

**TABLE 3:** Mechanical parameters of impact surfaces

Parameters	Rubber	Nylon	Foam
Density(kg/m^3^)	1,300	1,130	930
Young's modulus (MPa)	620	2,300	172
Poison Ratio	0.45	0.34	0.43

#### 2.1.1 Oil extraction

One day after impact, olive extraction was performed using Oliomio GOLD France laboratory extraction machine. This device consists of three parts: mill, mixer, centrifuge, and its application was extract oil on a laboratory scale. The extracted oils were stored in black glass at 4℃ (Pereira Et al., 2002).

#### 2.1.2 Evaluation of qualitative characteristics

Factors such as free fatty acidity (FFA), peroxide value (VA), and spectrophotometric indexes (K232, K270) of olive oil samples were determined in accordance with EU standard (EEC / 2565/91). The amount of chlorophyll and carotenoids was determined by Mongoose–Mosquera (1991) method using UV/Vis spectrometer T80 + PG Instrument at two wavelengths of 670 and 470 nm, respectively.

Extraction of phenolic compounds using methanol, acetonitrile, and ‐n hexane solvents after centrifugation and vacuum distillation (Eppendorf Concentrator plus Germany model) was performed according to the method of Pirisi et al., 2000. The amount of total phenol was determined by the Folin–Ciocalteu method at a wavelength of 760 nm using a spectrophotometer, and finally, the concentration of total phenol was calculated according to the standard gallic acid (mg GAE / kg oil).

Total flavonoid was measured according to method Du et al., 2009 using a spectrophotometer at 506 nm, and total flavonoid concentration was calculated according to the catechin standard as mg catechin / kg oil. This study was conducted in a completely randomized design with three replications as factorial. Statistical analysis was performed, and average comparison was done using Tukey's test.

### 2.2 Drop test and bruise phenomenon

The drop test was used to measure the amount of bruise volume caused by the collapse of the sample on the surface. Five olive samples of each class and three materials of rubber, foam, and nylon of surface were prepared. The olive sample drop on the surface from 1m height and the rebound height of the sample was obtained using the graded board which was placed behind the impact site. The impact energy was calculated using Equation 4 for each sample.

(4) $$E=mg\left(h_{i}‐h_{f}\right)$$

in which *E*, *m*, *g*, *h_i_*_,_ and *h_f_* were absorbed energy (J), mass (kg), gravity (9.81 m/s^2^), primary drop height (*m*), and rebound height (*m*), respectively.

**FIGURE 4:**
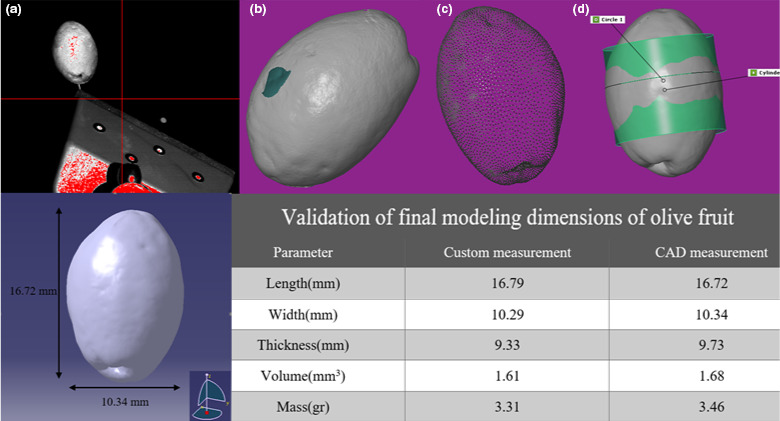
Process of reverse engineering method

The output of single point load cell was plotted as a force–time diagram in Excel software (Figure 4). Impact force was measured using a single point load cell (Model PW6CMR, HBM Inc., Marlborough, A, USA). The accuracy of force measurement and system sampling rate were 0.1 N and 10,000 Hz, respectively. The output load cell was isolated to the input strain gauge module (Model ADAM 3,016, Advantech Inc., Milpitas, CA, USA) in a data acquisition unit. A miniature circuit (Model BKN 1P C10A, LSIS Co., Ltd, South Korea) was placed between the power supply and the strain gauge module to protect the system during overload. The improved signal was inserted into a multifunctional USB module (Model USB‐4711A, Advantech Inc).

Due to the sign of bruise phenomenon of fruits can be observed after 24 hr, the bruise volume was measured by a digital caliper. After 24 hr, the damaged part of the olive fruit was completely discolored (Du et al., 2019; Jiménez‐Jiménez et al., 2013; Yousefi et al., 2016). The bruise volume was obtained using Equation 5.

(5) $$\text{BV}=\frac{\pi d}{24}\left(3w_{1}w_{2}+4d^{2}\right)$$

in which *w*_1_, *w*_2,_ and *d* were the length, width of the bruise region, and the maximum depth of the bruise area, respectively.

### 2.3 Reverse engineering and solid simulation process

To achieve more accuracy to solve finite element issues, the geometry of the modeled object must be very similar to the actual model (Celik et al., 2017; Fargnoli et al., 2012). Agricultural products have a heterogeneous and nonuniform shape that simple modeling cannot be used to obtain actual geometry. Hence, many researchers used reverse engineering techniques to produce the geometry of model (Celik et al., 2017; Celik et al., 2017; Du et al., 2019; Zhao et al., 2019). In this study, 3D optic scanning Shining (5 MP industrial cameras, resolution 0.04–0.16 mm, and volumetric accuracy 0.01 mm) was used (Figure 4a). In order to reduce the obtained cloud points error of the captured image caused by low camera precision, very low vibration, and reflected light, the GOM INSPECT, GEOMAGIC software was used to edit the captured images.

Edits include removing spark surfaces and smoothing surfaces (Figure 4b), reducing compressed cloud points (Figure 4c), and adjusting to the proper alignment axis (Figure 4d). The personal computer (RAM: 16GB, graphics: 4GB, Processor: Intel Core i7‐3.6 GHz) was used. In the next step, the edited points cloud was transferred to CATIA software and converted to a solid model using automatic modeling. Finally, the length, width, and thickness of modeled olive were compared with the actual shape and acceptable accuracy was observed.

### 2.4 FEA procedure

The finite element method is a numerical method for finding an approximate solution of the variable field distribution in the issue (Puri & Anantheswaran, 1993; Shen & Kushwaha, 1998). The formulated finite element method is the basis of a coordinate system and expresses the relation of each element. The local coordinate system of each element is to define the entire area of issue. In each element, it is possible to express the displacement functions simply in the form of polynomial interpolation in terms of the displacement values in its nodes based on its local coordinate system (Equation 6) (Wong et al., 2002).

(6) $$U^{h}\left(x,y,z\right)=\sum_{i=1}^{\text{nd}}N_{i}\left(x.y.z\right)d_{i}=N\left(x.y.z\right)d_{e}$$

In *h*, *n_d_*, *d_i_*_,_ and *d_e_* were quantity, the number of nodes forming the desired element, the displacement vector of the node for *i* node, and the displacement vector of the entire nodes, respectively. By solving the finite element equation of system, the displacement of the entire nodes achieves, and then, the stress and strain in each element will be obtained.

(7) $$d_{i}=\left[\begin{array}{c} u_{i}\\ v_{i}\\ w_{i} \end{array}\right]$$

(8) $$d_{i}=\left[\begin{array}{c} d1\\ d2\\ \vdots \\ dn_{f} \end{array}\right]$$

That *u_i_*, *v_i_*, and *w_i_* were the displacement component of the X, Y, and Z directions, respectively.

**FIGURE 5:**
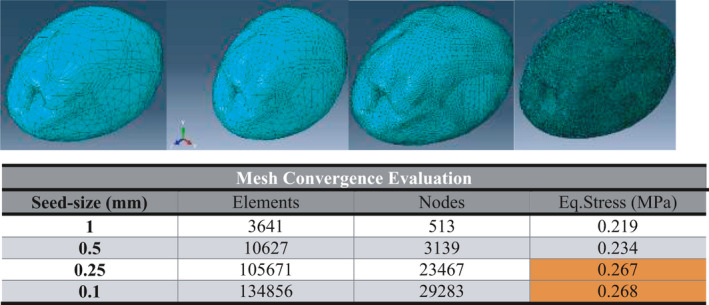
Determination of proper meshing size

The viscoelastic properties were extracted from the olive fruit stress relaxation test and then utilized for the simulation by ABAQUS software. For this purpose, Visco solver was used for the viscoelastic and time‐dependent solutions. Boundary, initial, and loading conditions play an important role in simulation. For plate‐to‐olive contact, surface‐to‐surface contact with a friction coefficient of 0.3 was considered. Tie constraint was used to prevent movement and deformation at the boundary between the olive and its kernel. Due to the fall height, velocity of olive fruit (three different angles of 0°,45°, 90°) was determined in the y direction (Figure 5). Literature of different fruits was presented as increasing drop height, the value of produced stress, and energy were increased (Gao et al., 2018, Celik, 2017, Li and Thomas, 2014, Jiménez‐Jiménez et al., 2013, Yousefi et al., 2016, Stopa et al., 2018). Hence, in this study a height of 1m was used for experimental and simulation. It should be noted that obviously, it is impossible to control the angle of olive drop during harvest and postharvest operation, but the damage can be reduced by adjusting the angle of impact surface. In addition, the gravity of 9,810 mm/s^2^ in the y direction was applied. The issue was solved dynamic explicit in 10 ms.

Finite element analysis is a process in which the mesh surface geometry is subdivided into smaller parts, then loads and boundary conditions are applied to these elements, and finally, the matrix equations are solved (Dennis et al., 2005). Theoretically, more number of used elements of the model leads to the results of actual behavior. However, analysis time has a direct relation with the number of elements (Dintwa et al., 2008) For this reason, meshing is one of the most important steps for simulation. In this study, the converge technique of results was used for appropriate mesh size. This technique was recommended by researchers (Celik, 2007, Souza et al., 2018) Therefore, the number of different seeds was utilized to obtain the result (stress) and eventually best mesh size was chosen (Figure 5). The best size was related to 0.50 mm of seed size that leads to 105,671 number of elements and 23,467 nodes. In order to the meshing olive flesh of olive, kernel, and impact surface the structure of free, C3D4 and C3D4 were applied, respectively.

## 3 RESULTS AND DISCUSSION

### 3.1 Simulation of drop test

**FIGURE 6:**
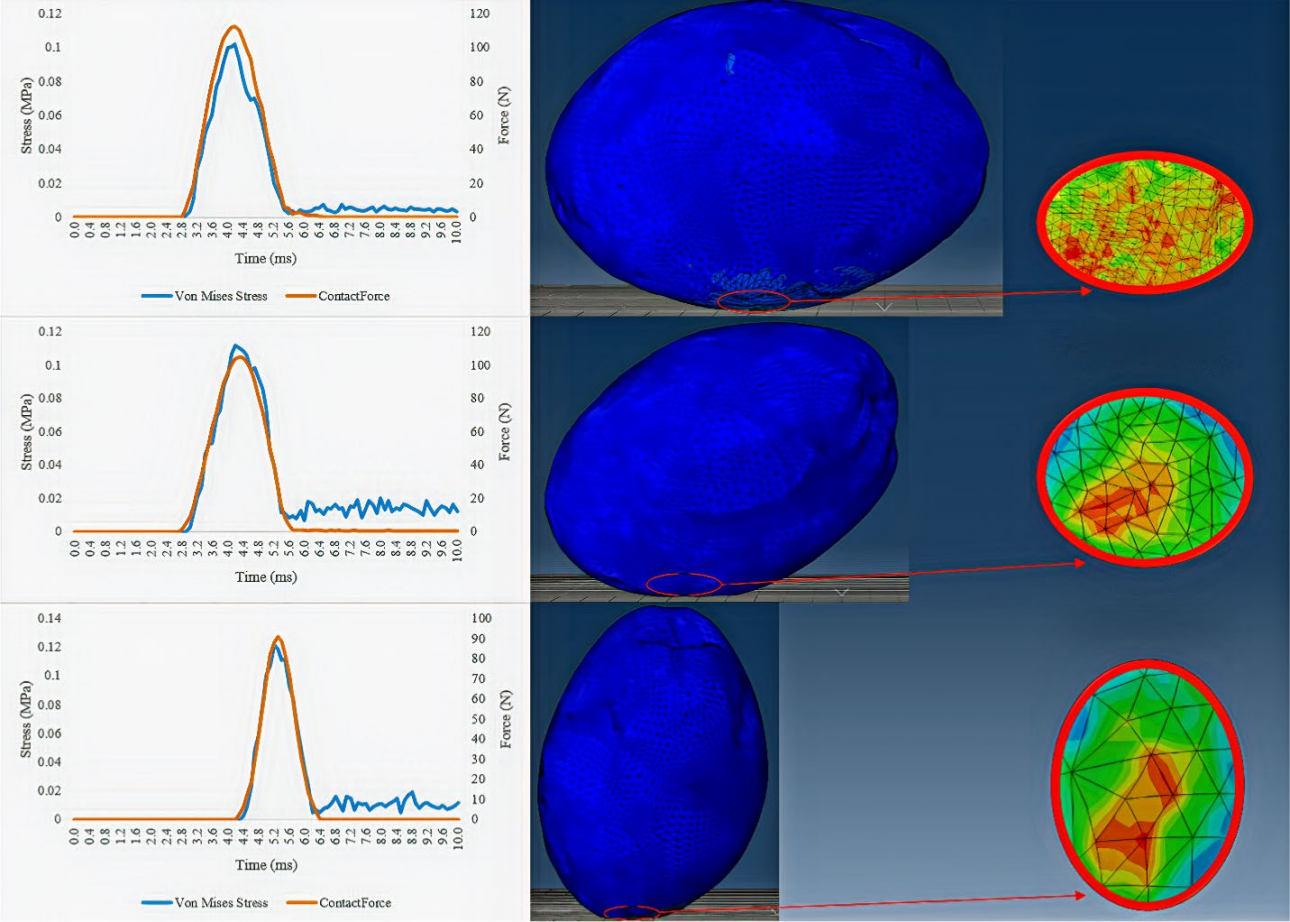
Stress distribution and contact force of simulation

Preprocessing steps of drop test for all classes were performed, and after processing, the numerical and printout results were stored. Figure 6 shows the results of the drop test (Oily olive on the rubber surface) in three modes of 0°, 45,° and 90° at the impact moment. The maximum produced stress of the impact for 0°,45°, 90° was 0.1, 0.112, and 0.122 MPa, respectively. Based on the results, when the fruit impacted the surface horizontally (0 °), less stress was produced than vertical situation (90 °). Similarly, experiments of pear (Yousefi et al., 2016), kiwifruit (Du et al., 2019), Lycium barbarum (Zhao et al., 2019) were demonstrated the generated stress enhanced with increasing angle of impact from 0° to 90°.

By contrast, as the angle increased (which leads to less contact surface), the contact force decreased. For instance, about the mentioned olive class, the maximum contact force of mentioned olive class at angles of 0°, 45°, and 90° was 112.3, 105.2, and 91.2 N, respectively. By contrast, as the angle increased (which leads to less contact surface), the contact force decreased. After impact to the surface, the olive entered the rebound stage and contact force became zero. However, due to the propagation of elastic stress waves, the stress did not become zero and the stress distribution across the olive fruit occurred. Also, the contact area for olive samples with impact angles of 0°, 45°, and 90° were 0.3 mm^2^, 0.22 m^2^, and 0.15 mm^2^, respectively.

Tables 4, 5, and 6 present the maximum amount of internal energy, total energy, stress, and contact force at the impact moment of olive samples with rubber, nylon, and foam surfaces, respectively. For each surface, 27 simulations and totally 81 simulations were performed. In all simulations, the amount of internal energy, total energy, and produced stress of the Oily cultivar was greater than the other two varieties. For example, in simulation No. 12 of rubber surface (olive variety: Oily, ripening stage: unripe, impact angle: 90 °) had the highest internal energy (152.3 mJ), total energy (154.2 mJ), and stress (0.351 MPa).

**TABLE 4:** Simulation results of drop test (rubber surface)

Variety	Ripening Stage	Angle of impact	Max. Internal Energy (mJ)	Max. Total Energy (mJ)	Max. Equivalent Stress (MPa)	Max. Contact Force (*N*)	Number of Simulation
Yellow	Unripe	0°	145.3	146.5	0.308	8.5	1
		45°	147.2	148.7	0.320	6.2	2
		90°	147.8	149.1	0.329	3.7	3
	Semiripe	0°	117.2	118.7	0.150	25.5	4
		45°	119.2	121.3	0.161	21	5
		90°	119.4	120.8	0.172	13.8	6
	Ripped	0°	74.6	76.8	0.052	86.1	7
		45°	76.1	77.2	0.061	70.4	8
		90°	76.8	78.1	0.065	61.3	9
Oily	Unripe	0°	149.2	151.3	0.330	10.7	10
		45°	150.7	151.7	0.348	15.4	11
		90°	152.3	154.2	0.351	17.9	12
	Semiripe	0°	128.4	129.7	0.187	49.3	13
		45°	130.7	131.3	0.201	41.3	14
		90°	132.8	134.1	0.212	33.8	15
	Ripped	0°	94.2	95.3	0.1	112.3	16
		45°	94.6	95.9	0.112	105.2	17
		90°	95.1	97.2	0.122	91.2	18
Fishemi	Unripe	0°	138.1	139.4	0.266	15.1	19
		45°	138.5	140.8	0.286	10.4	20
		90°	140.1	142.3	0.301	8.7	21
	Semiripe	0°	108.7	110.4	0.131	34.2	22
		45°	111.7	112.4	0.140	30.4	23
		90°	112.5	114.1	0.157	24.7	24
	Ripped	0°	67.4	68.4	0.044	90.1	25
		45°	68	69.7	0.052	81.3	26
		90°	68.2	70.2	0.060	70	27

**TABLE 5:** Simulation results of drop test (Nylon surface)

Variety	Ripening Stage	Angle of impact	Max. Internal Energy (mJ)	Max. Total Energy (mJ)	Max. Equivalent Stress (MPa)	Max. Contact Force (*N*)	Number of Simulation
Yellow	Unripe	0°	120.8	122.9	0.288	8.2	1
		45°	123.5	124.7	0.301	5.9	2
		90°	131.7	133.5	0.311	3.1	3
	Semiripe	0°	80.7	82.0	0.143	24.7	4
		45°	88.2	89.7	0.157	20.1	5
		90°	91.1	120.8	0.171	12.4	6
	Ripped	0°	50.6	53.4	0.039	84.3	7
		45°	57.3	59.7	0.047	69	8
		90°	61.2	61.8	0.050	59.1	9
Oily	Unripe	0°	132.7	134.8	0.331	8.7	10
		45°	140.3	141.7	0.337	14.2	11
		90°	144.3	145.2	0.342	16.3	12
	Semiripe	0°	90.2	93.1	0.173	47.8	13
		45°	91.1	93.9	0.191	41.1	14
		90°	91.5	95.1	0.201	31.5	15
	Ripped	0°	66.6	69.2	0.089	110.2	16
		45°	71.3	73.4	0.102	105	17
		90°	78.2	81.7	0.111	90.4	18
Fishemi	Unripe	0°	107.4	110.2	0.262	14.1	19
		45°	114.3	116.7	0.271	8.3	20
		90°	120.5	123.7	0.284	7.2	21
	Semiripe	0°	70.1	71.8	0.120	31.7	22
		45°	74.8	75.3	0.132	30	23
		90°	81.2	83.7	0.146	22.4	24
	Ripped	0°	42.9	44.4	0.031	87.3	25
		45°	50.7	52.8	0.035	78.4	26
		90°	55.3	59.7	0.047	66.7	27

**TABLE 6:** Simulation results of drop test (foam surface)

Variety	Ripening Stage	Angle of impact	Max. Internal Energy (mJ)	Max. Total Energy (mJ)	Max. Equivalent Stress (MPa)	Max. Contact Force (*N*)	Number of Simulation
Yellow	Unripe	0°	110.5	112.3	0.266	7.5	1
		45°	119.1	122.4	0.281	5.1	2
		90°	119.3	122.9	0.290	2.6	3
	Semiripe	0°	66.3	68.3	0.133	21.7	4
		45°	71.2	74.1	0.141	17.6	5
		90°	76.3	79.5	0.155	10.7	6
	Ripped	0°	31.5	33.7	0.024	78.4	7
		45°	37.6	38.4	0.033	62.7	8
		90°	41.2	43.8	0.049	55.7	9
Oily	Unripe	0°	118.4	122.1	0.291	6.8	10
		45°	119.4	123.7	0.299	11.7	11
		90°	121.5	124.8	0.313	13.8	12
	Semiripe	0°	73.4	75.3	0.157	44.3	13
		45°	80.6	82.7	0.176	37.9	14
		90°	81.3	83.6	0.190	26.6	15
	Ripped	0°	50.8	52.7	0.088	114.5	16
		45°	51.3	54.9	0.104	99.8	17
		90°	56.1	58.7	0.112	85.6	18
Fishemi	Unripe	0°	101.8	104.3	0.255	11.1	19
		45°	103.7	105.8	0.261	6.8	20
		90°	111.2	113.9	0.272	5.5	21
	Semiripe	0°	59.9	62.3	0.117	27.6	22
		45°	66.1	69.7	0.122	26.8	23
		90°	69.2	71.3	0.134	19.3	24
	Ripped	0°	23.3	26.7	0.008	81.5	25
		45°	30.8	34.6	0.018	73.6	26
		90°	35.7	38.2	0.026	61.9	27

Regarding the results of the surface impact with ripped olive samples not only the generated stress decreased but also the contact force impact declined. The reduction of mentioned factors of Yellow and Fishemi varieties was more than Oily cultivar in the semiripe and ripped stages. It seems the effect of fruit water had a significant effect on fruit resistance and it was similar to previous research of the other fruits (Du et al., 2019; Yurtlu & Erdoğan, 2005; Zhu et al., 2016). Although the amount of olive water decreased with increasing olive ripening, the enhancement of oil (which generated in the ripening period) can partially increase the resistance of the olive flesh to impact. Hence, the amount of internal energy absorption and produced stress increases.

The stress, internal energy, total energy, and contact force caused by nylon and foam surface were lower than rubber. The lowest amount of internal energy, total energy, and stress was related to simulation No. 25 of foam surface (olive variety: Fishemi, ripening stage: Ripped, Impact Angle: 0°) and the amount of mentioned indicates were 23.3 mJ, 26.7 mJ, and 0.008 MPa, respectively. Since the density and elastic modulus of foam were lower than nylon, the amount of produced energy (that has direct related to the bruise rate) was less. Comparably, Zhao et al. investigated the bruise volume at the impact moment of Lycium Barbarum fruit with the wood, nylon, and foam boards and reported the lowest bruise rate was related to the impact of fruit and nylon surface (Zhao et al., 2019).

**FIGURE 7:**
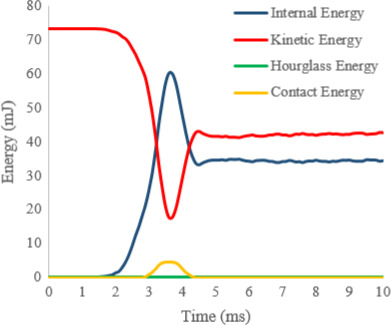
Energy evaluation of the typical simulation

Energy analysis was performed to validate the simulation model for all classes and types of impact surfaces. Figure 7 shows the amount of internal, kinetic, contact, and hourglass energy for a sample of olive in the impact moment with rubber surface. During the drop of olive, the potential energy was converted to kinetic energy, and after the collision, the absorbed energy (internal energy and contact energy) was observed. Hourglass energy, which is an important factor in determining the finite element accuracy, was investigated. One of the limitations of the large meshing is the hourglass phenomenon which leads to meaningless results (Tsang & Raza, 2018). In the case of reduced first‐order and second‐order elements, the reduction in mesh size can decrease the occurrence of the hourglass phenomenon. According to the researchers' suggestion, hourglass energy should not exceed 5%–10% of internal energy (Celik, 2017; Du et al., 2019). In this study, by comparing the hourglass energy and internal energy, it can be concluded that meshing and finite elements were acceptable.

### 3.2 Evaluation of bruise volume

**FIGURE 8:**
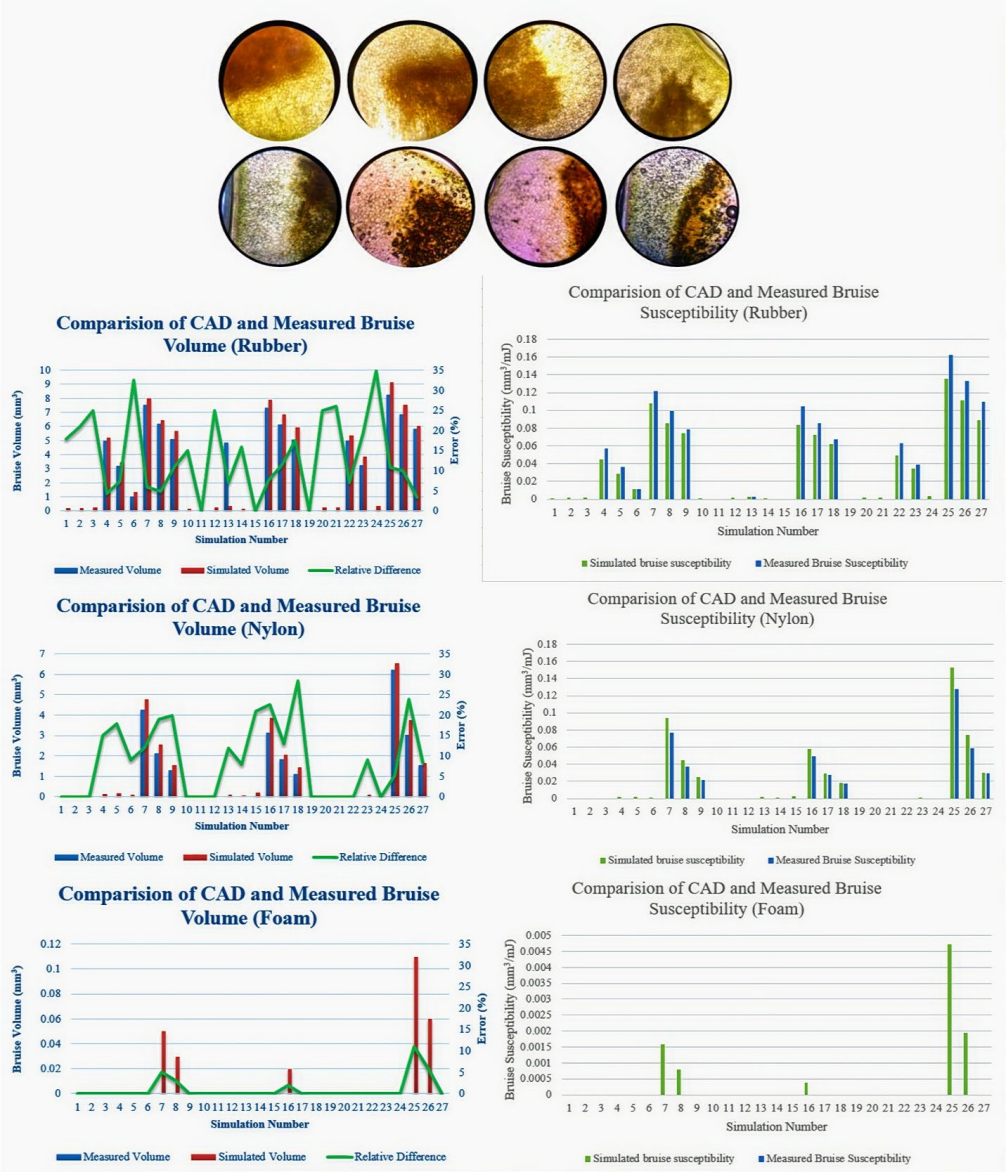
Results of measured and simulated bruise volume and bruise susceptibility. *Note*: Error is of relative change calculated from the change between the measured and simulated accepted values, and dividing by the measured value

The amount of bruise volume of olive samples in the drop test was determined. Depending on the olive cultivar, bruising was occurred beneath of skin. It can be seen that impact caused damage to olive flesh's endocarp. By applying impact to the olive, of the mesocarp layer which is elastic and firm, it compresses the endocarp layer between itself and the kernel (Rapoport et al.,^2016^). When the impact force was stopped, the mesocarp layer returned to its original condition, and the spoiled layer broke off from its transverse section. The area of the impact at the microscopic dimension was clearly visible using a microscope (Figure 8). Obviously, the area of the bruise is color distinct from other parts of the surface. The damaged cellular tissue was almost corrupted, and in some areas, the cell membrane was ruptured and the cellular fluid was released which causes discoloration and bruising.

The shape of the bruising was different in the experiment samples. In Oily variety, the shape of bruise was spherical, but the bruise shape of yellow and Fishemi cultivars was elliptical. The damaged tissue of Oily samples was more concentrated while the damage tissue of Fishemi and Oily varieties was more elongated. In previous research, the shape of bruise area has been reported both in the elliptical (Saracogluet al., 2011) and in the spherical that similar to apples and pears (Blahovec & Paprštein, 2005; Opara, 2007). This could be due to the differences in physical properties especially the sphericity of the three varieties. Also, the larger kernel, the smaller thickness of flesh, and the mass of olive probably were another reason for the larger bruise volume.

The maximum bruise volume was occurred when the Fishemi olive impact the rubber surface horizontally (0 °) (8.26 mm^3^). In the drop test of olive fruit on rubber and nylon surfaces, several flesh of olive was undamaged and nonbruised. However, in all of the tests related to foam surface, no bruising was observed. In addition, the amount of simulated bruise volume was calculated for all tests. The amount of stress in areas of olive fruit which was more than the bioyield stress point during impact moment can be considered as a bruise phenomenon (Celik, 2017). For this purpose, the area transferred to the CAD software and the volume was obtained.

Finally, the amount of simulated and measured bruise volume were calculated. Maximum amount of bruising was happened on the rubber surface (Fishemi variety, angle of impact: 0 and level of maturity: ripped). These results were shown the angle and maturity level of olive had considerable effect on the bruise volume. Also, the maximum error of rubber, nylon, and foam surface tests was 35%, 28.5%, and 11%, respectively. Error is a special case of the percentage form of relative change calculated from the change between the measured and simulated accepted values and dividing by the measured value. Du et al. (2019) performed a study on the bruise volume of kiwifruit and reported that the maximum and minimum errors were 17.1 and 3.8, respectively. Although in this study the maximum error was greater than their study, the minimum error was 0%.

By availability of the internal energy and bruise volume, the bruise susceptibility was investigated. The maximum measured bruise susceptibility of olive impact with rubber, nylon, and foam surfaces was 0.162, 0.127, and 0 m^3^. J^‐1^ and the simulated bruise susceptibility was 0.135, 0.152, and 0.004 m^3^. J^‐1^, respectively. It should be noted that although the bruise volume of the olive samples was measured after 24 hr of the drop test, probably the bruise volume could be more (depending on the mechanical and chemical properties of the olive cultivars) after several hours.

### 3.3 Evaluation of chemical characteristics

#### 3.3.1 FFA and PV

**FIGURE 9:**
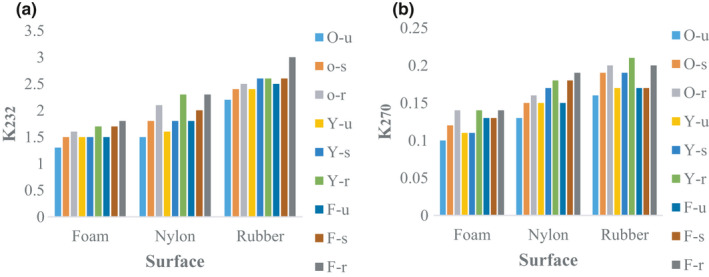
Result of free fatty acid and peroxide value of olive oil samples. (Note: The abbreviations are O: Oily, Y: Yellow, F: Fishemi, u: unripe, s: semiripe, r: ripped)

Comparison of means showed the free fatty acid of the oil samples was increased when dropped on the nylon and rubber surface. The maximum fatty acid content was 0.59% (Fishmi ripe), which had harvest by Neoprene material (Figure 9a). Due to the increase of FFA, the volatile acids of olive oil such as acetic acid and butyric acid increase (Ciafardini & Zullo, 2018). Hence, it causes a musty odor in the extracted oil, which reduces the desire to consume olive oil.

Similarly, the mechanical damage caused by the drop increased the amount of peroxide (Figure 9b). The maximum and minimum peroxide values were related to extracted oils from Fishemi‐ripped and yellow‐unripe samples, respectively (harvested by Neoprene IH—harvested by Manjid IH). It seems that the impact increased the oxidation of unsaturated fatty acids and increased the activity of the enzyme lipoxygenase (lox), which increased the amount of peroxide (Zhang et al., 2020).

#### 3.3.2 K232 and K270 coefficients

**FIGURE 10:**
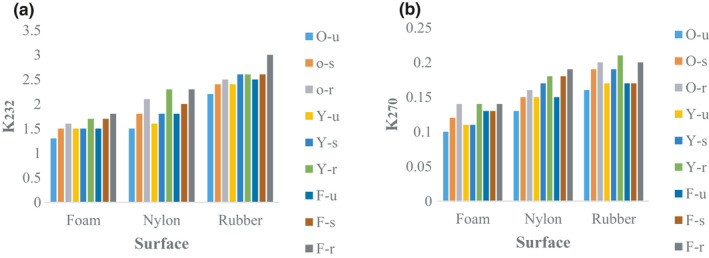
Result of K232 and K270 of olive oil samples

Although mechanical damage of samples increased the amount of k232, the results did not show a statistically significant difference with the blank samples (Figure 10a). In contrast, the k270 of olive oil samples of yellow varieties was significantly different from other cultivars. Also, the lowest k270 of olive oil was related to the Oily unripe sample (Figure 10b).

#### 3.3.3 Total chlorophyll, carotenoids, phenol, and flavonoids

**FIGURE 11:**
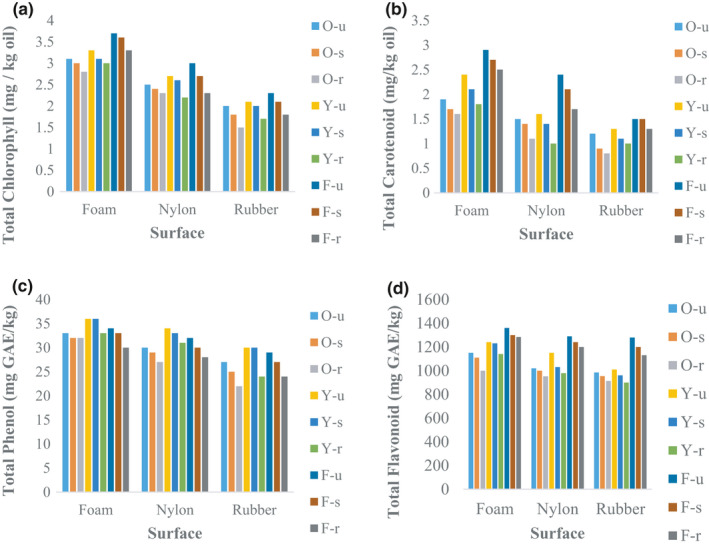
Results of Total chlorophyll, carotenoids, phenol, and flavonoids of olive oil samples

When the olive samples drop on the rubber surface, the amount of chlorophyll and carotenoids of extracted oil samples significantly decreased compared to the blank samples (Figure 11a,b). The minimum chlorophyll content was related to the Oily ripped sample‐contact with the rubber surface (1.5 mg/kg oil). When olive dropped on the rubber surface, the proportion of chlorophyll converts to pheophytin, which can be a factor in carotenoid reduction (Mraicha et al., 2010).

Although there was no statistically significant difference in the affected samples compared to the blank samples, the amount of phenol and flavonoids of extracted oil was decreased (Figure 11c,d) Maximum amount of total phenol and flavonoids were related to the yellow variety, while previous studies also claimed that the total yellow phenol and flavonoids were more than the oily cultivar (Kharazi, 2008). Mechanical damage during the impact process increased the oil oxidation process, which reduced total phenolics.

## 4 CONCLUSION

The results showed that the reverse engineering method was a proper method for obtaining the geometry of fruit. The drop test was simulated for all classes on three different surfaces. The maximum and minimum produced stress, the internal energy, and total energy were related to unripe Oily and ripped Fishemi varieties, respectively. Furthermore, the angle of impact had a considerable effect on the amount of stress, energy, and contact force. As the impact angle increased, the contact force decreased and the stress increased. In the next step, the hourglass energy factor was used to validate the simulated energy. Based on the results and the comparison of the hourglass energy with the absorbed energy, it was concluded that the meshing size and finite element were performed properly.

Finally, the bruise volume and the bruise susceptibility of the simulation method were obtained and were compared with the measured values. In most of the experiments, the measured bruise volume was zero, approximately. According to the simulated and measured results, it can be claimed that the finite element method was a reliable method for estimating the amount of produced stress and energy in different conditions and also a reliable method for predicting the amount of bruise volume and bruise susceptibility.

Mechanical damage during the drop process increased the free fatty, peroxide, K232, and K270 values of extracted oil samples. Most of the chemical changes were related to the Fishemi variety. Although damaged olive had more levels of FFA and PV in this experiment, it had the proper quality for oil extraction. Damaged olive samples when drop to the rubber surface, the chlorophyll, carotenoids, phenol, and flavonoids of extracted olive oil compared to the blank samples were significantly reduced. This study is useful for developing the process of olive sorting in food industries as well as transportation. Further research can lead to optimize the postharvest process of olive systems. Finally, improved design of the olive postharvest process system's lead to decrease bruise volume of olive. Therefore, high‐quality extra virgin olive oil can be achieved.

## CONFLICTS OF INTEREST

The authors have declared no conflicts of interest in this article.

## AUTHOR CONTRIBUTIONS

**Mahdi Rashvand:** Conceptualization (equal); Software (equal); Writing‐original draft (equal). **Abbas Akbarnia:** Project administration (equal); Supervision (equal). **Rouzbeh Abbaszadeh:** Resources (equal); Software (equal). **Davoud Karimi:** Methodology (equal). **Ali Jafari:** Validation (equal); Visualization (equal).

## ETHICAL APPROVAL

This study does not involve any human or animal testing.

## Data Availability

The authors confirm that the data supporting the findings of this study are available within the article.
